# The dynamics of dyadic interactions between people of different ethnicities depend on their identification with all humanity

**DOI:** 10.1038/s41598-022-25905-9

**Published:** 2022-12-15

**Authors:** Katarzyna Hamer, David López Pérez, Marek Drogosz, Henryk Goworek

**Affiliations:** 1 Institute of Psychology of Polish Academy of Sciences, Jaracza 1, 00-378 Warsaw, Poland; 2 SWPS University of Social Sciences and Humanities, Chodakowska 19/31, 03-815 Warsaw, Poland; 3 Maria Sklodowska-Curie Medical Academy, Aleja Solidarności 12, 03-411 Warsaw, Poland

**Keywords:** Psychology, Human behaviour

## Abstract

Individuals who have the disposition to identify with all humanity declare feeling close to people all over the world, caring about them, and perceiving them as an ingroup. However, never before were such declarations verified by measures of intergroup attitudes less direct than questionnaires, such as approach/avoidance tendencies or dynamical systems methods. Since individuals with higher dispositional identification with all humanity (IWAH) perceive people all over the world as ingroup members, we expected differences in the dynamic of inter-ethnic interactions (spatial distance, coordination, coupling, and leading), depending on a participant’s level of IWAH. 227 participants fulfilled the IWAH scale, and those with the highest and lowest scores took part in a laboratory study, performing a task in inter-ethnic dyads. For the first time, an approach that combines a state-of-the-art tracking algorithm with a dynamical systems method was applied in such a context. Our results showed that those higher in IWAH kept a smaller distance from a partner, took a more leading role, and showed better coordination than those lower in IWAH. We found a similar trend for coupling. The results demonstrated the importance of IWAH for inter-ethnic relations and how it may shape non-verbal behaviors. Limitations are discussed.

## Introduction

Individuals who have the disposition to identify with all humanity feel close to people worldwide, care about them, and perceive them as an ingroup. Research shows that they care more for human rights and humanitarian needs^1–3^, declare more willingness to include people from different ethnic groups into friends groups, show less dehumanization and islamophobia, and are more prone to helping people from other countries^2,4^ than those who weakly identify with all humanity. However, all these results were obtained with questionnaires and are mostly declarations. In this paper, we use dynamical systems approach together with more implicit measures of attitudes to research issues from intergroup relations, personality, and social psychology. To our knowledge, it is the first time such an approach has been implemented in the context of identification with all humanity (IWAH). It allowed us to look more precisely into the dynamics of dyadic interactions of individuals with different levels of IWAH with people of different ethnicities.

In dynamical systems, a team is viewed as a system of coupled elements that interact over time^5^. Since individuals with higher dispositional identification with all humanity perceive people all over the world as ingroup members, we expected differences in the dynamic of inter-ethnic interactions, depending on a participant’s level of IWAH. In this paper, for the first time, an approach that combines a state-of-the-art tracking algorithm with a dynamical systems method was applied in such a context, using indirect measures of intergroup attitudes, such as spatial distance, coordination, coupling, and leading.

### Approach/avoidance tendencies as indirect measures of attitudes toward ingroup and outgroup members

Numerous studies describe differences in attitudes and behaviors towards own ingroup compared to outgroups, showing ingroup favoritism and outgroup derogation. Attitudes toward ingroup and outgroups can be manifested by approach/avoidance tendencies^6–8^. Many studies showed that people tend to spontaneously approach what is liked and avoid what is disliked (see, e.g.^9^). Paladino and Castelli^7^ demonstrated that social categorization into ingroup and outgroup involves “the immediate and spontaneous activation of specific motor tendencies, so that individuals are predisposed to approach ingroup members rather than outgroup members, and are more likely to avoid outgroup members." (p. 767). These approach-avoidance behaviors are considered spontaneous and outside of participants' awareness, bypassing intentional control and social desirability concerns^7^. Therefore, they could serve as an indirect test if people higher in IWAH indeed treat a partner of different ethnicity more as an ingroup member than people lower in IWAH do. Indirect measures are often considered as less influenced by self-presentation tendencies compared to direct measures (e.g., self-report^8,10^), which gives them an advantage over direct measures (questionnaires) used so far to assess attitudes of people with different levels of IWAH.

Spatial distance is a measure of approach/avoidance tendencies successfully used to assess group stereotyping, prejudice, and discrimination in numerous previous studies (e.g.^11–15^). Results of these studies confirmed that the distance kept from outgroup members is bigger than from ingroup members. Therefore we suspect that if those high in IWAH truly include people of different ethnicities in their own ingroup, the spatial distance they keep while interacting during performing a task with such a person should be smaller than for those lower in IWAH.

The situation of working on a task in a dyad allows us to observe not only spatial distance and avoidance tendencies but also additional behavioral markers of attitudes and do so in time. Social interactions require individuals who work together to coordinate intentions and actions^16^. From a dynamical systems perspective, complex behavior is nested in sequences of previous and future behavioral events, and the degree to which previous behavior determines the subsequent behavior can differ^17^. Within a dyad, two dynamical systems interact with each other and form a synergy to work towards the same goal (e.g.^18,19^). In this context, they work as a functional grouping of systems that work together and self-organize, trying to build something together. Within this synergy, changes in one component are compensated for by variations in other components to preserve the functional goal of the synergy. In this context, participants working in dyads need flexibility in their complex behavior to handle and adapt to the task demands. We suspect that given the task at hand (building construction together), both groups of people, either with low or high IWAH, will be to some degree coordinated with their task partner. Still, the quality and complexity of this coordination will differ among groups, with the higher-IWAH displaying a more complex and better interaction with a partner than lower-IWAH (as measured by coordination, coupling, and leading) participants. Coupling can be defined as these observable patterns that show a close temporal similarity or complementarity of behaviors: "Coupling simply means that the processes have some form of interaction with each other"^5^ (p. 3). Coordination can be seen when behaviors show close temporal coupling. We operationalize cooperation and active role during a task as leading: relative temporal relationships between behaviors, such as of two individuals in a joint task where one of them is more active and the other mimics or follows the movements after a certain delay. We believe that less coupling and less coordination between partners, as well as less cooperation and a more passive attitude during a joint task (less leading), can be perceived as non-verbal markers of avoidance tendencies; therefore, it should be more common among a lower-IWAH group.

Thus, we formulated hypotheses that during performing a joint task (building a LEGO construction), while interacting with a person of different ethnicity, people higher in IWAH will keep a smaller distance to a partner (H1), be more active by taking a more leading role (H2), show better coordination (H3) and coupling (H4) with a partner than those lower in IWAH.

We tested these hypotheses in a 2-stage study: first, students fulfilled questionnaires evaluating their level of identification will all humanity, and then those with its highest and lowest levels took part in the laboratory study, where they performed a 5-min task in an inter-ethnic dyad, followed by a short survey. Since Poland is ethnically a very homogenous country (see e.g.^20^), we picked Nigerian students to be lab assistants in dyads with Polish student participants due to a significant cultural distance (bigger than it would be, e.g., with Ukrainian assistants, etc.).

## Results

### Shyness, intergroup friendships, and evaluation of the LEGO task

First, we conducted analyses in order to check if the low- and high- IWAH groups differ in shyness or evaluation of the LEGO task. We found no significant differences in shyness: *t* (23.6) = − 0.496; *p* > 0.05 (*M *of high-IWAH group = 2.64; *M *of low-IWAH group = 2.79), task liking: *t* (24.8) = 1.05; *p* > 0.05 (*M *of high IWAH group = 3.56; *M *of low IWAH group = 3.25), satisfaction with the final result: *t* (16.14) = 0.98; *p* > 0.05 (*M *of high IWAH group = 3.75; *M *of low IWAH group = 3.5), nor how easy or difficult it was to reach an agreement about the LEGO task project:* t* (24.07) = 1.05; *p* > 0.05 (*M *of high IWAH group = 4.5; *M *of low IWAH group = 4.25)*.* Further, there were no differences between groups in frequency of having friends or acquaintances with skin color: *t* (22.55) = − 0.65; *p* > 0.05 (*M *of high IWAH group = 1.29; *M *of low IWAH group = 1.42), nor the closeness of this bond:* t* (20.97) = 1.62; *p* > 0.05 (*M *of high IWAH group = 2.43; *M *of low IWAH group = 1.75).

Next, we conducted several analyses to compare indices of interaction dynamics (spatial distance, leading, coordination, and coupling in dyads) between low- and high-IWAH groups.

### Dynamics of the interaction: spatial distance

**Figure 1:**
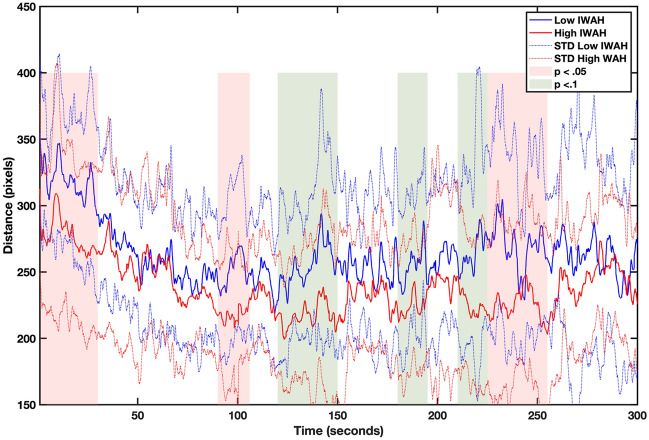
Average distance in pixels between participants and a lab assistant for the low-IWAH (blue) and the high-IWAH (red) groups. Differences in the distance were tested in blocks of 15 s. Significant differences (*p* < 0.05) between distances are highlighted as red-shaded areas while trends (*p* < 0.1) are highlighted as green-shaded areas.

DeepLabCut (DLC, for details, see Supplementary Materials B and C, and the method section) was able to track the six labeled body parts with high precision during the entire video with a training error of 1.92 pixels (*SD* = 0.09) and a test error of 4.11 pixels (*SD* = 1.96), which highlights the high accuracy of the algorithm (video resolution was 1280 × 720 pixels). We tested differences in pixel distance between a label point in the face (i.e., a nose) of a participant and a lab assistant and compared the average values between groups in blocks of 15 s. Figure 1 shows the average spatial distance between low-IWAH and high-IWAH participants during the interaction.

The results show that, on average, the low-IWAH group kept further away from a lab assistant during the task (*U* = 144; *p* = 0.026; Mean rank for high-IWAH = 11.50, Mean rank for low-IWAH = 18.50). Then, we compared all the blocks separately. Wilcoxon signed-rank tests showed significant differences (see Supplementary Table 1 for details) at the beginning and during some parts throughout the interaction (75 s in total, shadowed in red; see Fig. 1) as well as some statistical trends (60 s in total, shadowed in green; see Fig. 1). These results confirmed hypothesis 1.

To analyze the distance during an interaction in more detail, we studied the combined positions of participants and lab assistants, and the distance between them (see Supplementary Material E). Comparison between the horizontal position of the lab assistants and participants showed that the positions of lab assistants were rather constant in both groups, while the positions of participants differed: high-IWAH participants kept closer to their partner than the low-IWAH participants. We interpret these results as an indicator that distance-wise the differences were due to participants’ positions rather than the behavior of lab assistants.

### Dynamics of the interaction: leading

**Figure 2:**
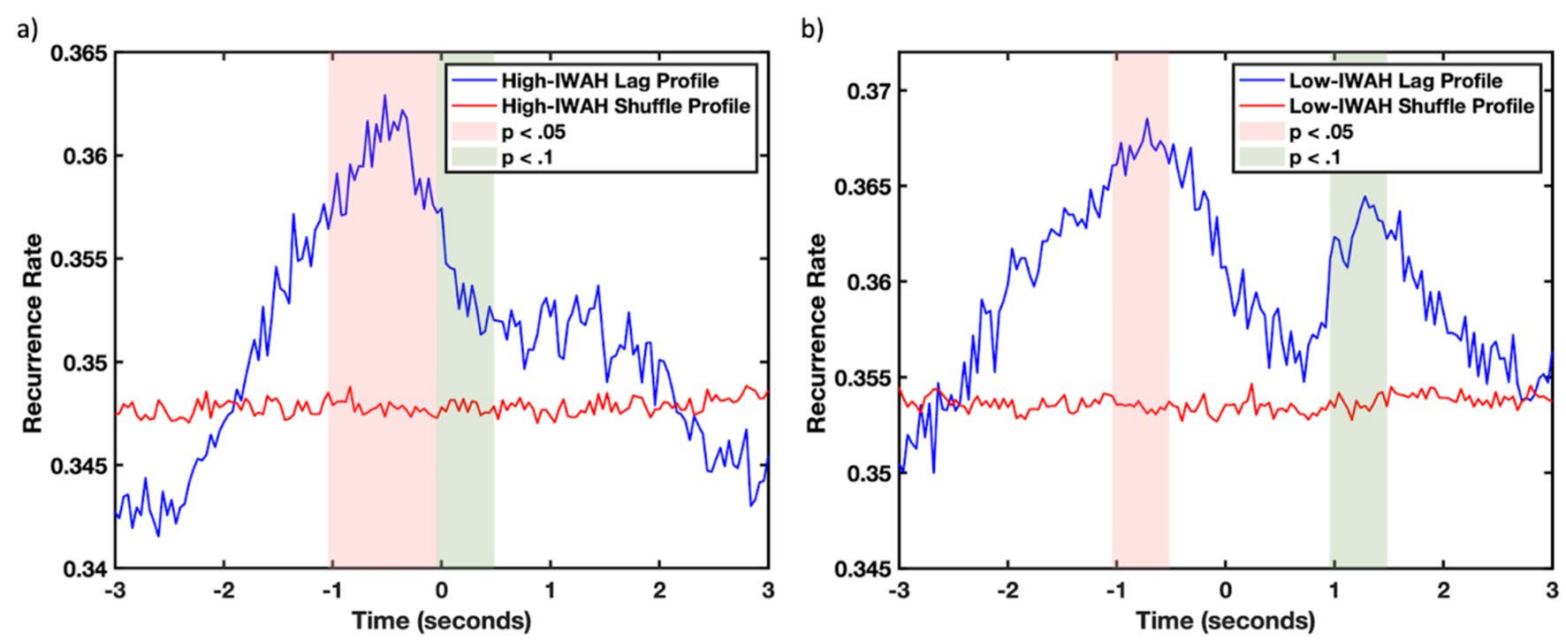
Average lag profiles between a participant and a lab assistant computed using diagonal-wise CRQA for the high-IWAH (**a**) and the low-IWAH (**b**) groups. The light red and light blue lines represent the standard deviation of mean normal and shuffled profiles, respectively. The red shaded area represents the time window where significant differences were found between the original (blue), and control shuffled profiles (red), while the green indicates areas reaching significance (trends). Recurrence rate values are normalized between 0 and 1.

Next, we categorized the movement extracted from DLC and applied Cross-Recurrence Quantification Analysis (CRQA; see the method section) to explore the dynamics of the interactions in both groups. The CRQA lag profile (Fig. 2) showed that all dyads were highly coordinated, where a peak of coordination arose in both groups at a maximum of − 720 ms for the low-IWAH group (Fig. 2a) and − 360 ms for the high-IWAH one (Fig. 2b). Negative values of the lag profile correspond to a participant leading the movements in interaction, while positive values are related to a lab assistant leading. In both groups, participants seemed to be leading the interaction, which was expected as lab assistants were instructed to let participants lead the interaction and step in only if a participant would be passive.

However, high-IWAH participants were leading longer and in a more stable manner (Fig. 2a), while we observed a drop in leading in the low-IWAH group (Fig. 2b). On the right-hand side of the lag profile (i.e., a lab assistant leading the interaction), there is a smaller peak arising for the low-IWAH group while this part is flatter in the high-IWAH group. The significant differences in this part of the profile suggest that participants in the low-IWAH group were less proactive during the interaction and a lab assistant had to occasionally take on a leading role during the interaction. This was not observed in the high-IWAH group.

Further comparisons in half a second steps with the shuffled version of the profile (Fig. 2a,b) showed that the effect of the movement coupling in the task as captured by cross-recurrence profiles is significantly different than the random-paired baseline level, and it cannot be attributed to the task itself. Particularly, in the high-IWAH group, Wilcoxon signed-rank tests showed significant differences between the lags from − 1 to − 0.5 s (*Z* = 2.50, *p* = 0.01) and from 0.5 to 0 s (*Z* = 2.31, *p* = 0.02), while a trend was present from lags 0 to 0.5 s (*Z* = 1.90, *p* = 0.05). In the low-IWAH group, however, we found only significant differences between the lags from − 1 to − 0.5 s (*Z* = 2.10, *p* = 0.03) and a trend from 1 to 1.5 s (*Z* = 1.77, *p* = 0.07). These results confirmed hypothesis 2.

### Dynamics of the interaction: coordination

**Figure 3:**
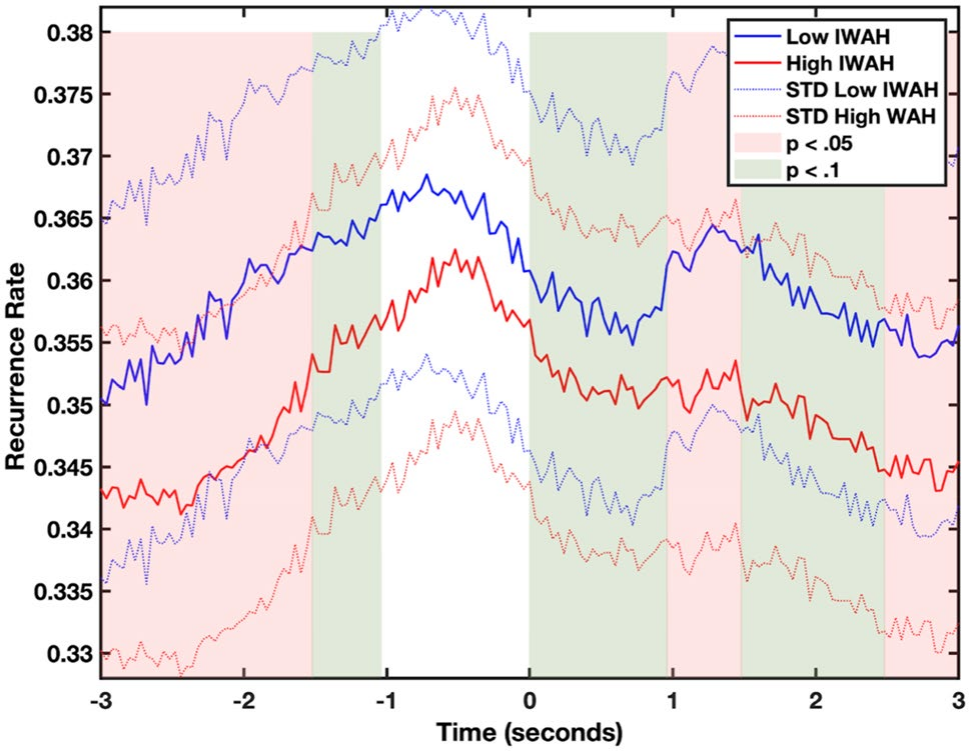
Cross-recurrence lag profiles between the movements of participants and lab assistants for the low-IWAH (blue) and the high-IWAH (red) groups. Differences in recurrence rate were tested in blocks of 0.5 s. Significant differences (*p* < 0.05) between the lag profiles are shadowed in red while trends (*p* < 0.1) are shadowed in green. Negative values in time indicate that a participant led the interaction, while positive values suggest that a lab assistant did. Recurrence rate values are normalized between 0 and 1.

We further compared the average lag profiles in order to find temporal differences between both groups. Figure 3 shows that at the time around the peak maxima, there are no significant differences between both groups, suggesting that both groups are coordinated during the interaction even if the recurrence rate maxima vary its position slightly. However, as we move from the peak maxima, there are significant differences in how both groups coordinate (see Supplementary Table 2 for Wilcoxon signed-rank tests full results). Next, following Nomikou et al.^21^, we further analyzed the lag profiles. Particularly, we found that the kurtosis of the high-IWAH group was higher (Kurtosis_HIGH_ = 2.20) than the low-IWAH group (Kurtosis_LOW_ = 2.13). Likewise, the skewness of the profile was higher in the high-IWAH (Skewness_HIGH_ = 0.22) than in the low-IWAH group (Skewness_LOW_ = − 0.01). Finally, the dispersion of the high-IWAH group was lower (Dispersin_HIGH_ = 1.86) in comparison to the low-IWAH group (Dispersion_LOW_ = 1.93). Altogether, it suggests that even if the recurrence rate between high-IWAH participants and a lab assistant is lower, the quality of the coordination was better in comparison to the low-IWAH one. These results confirmed hypothesis 3.

### Dynamics of the interaction: coupling

**Figure 4:**
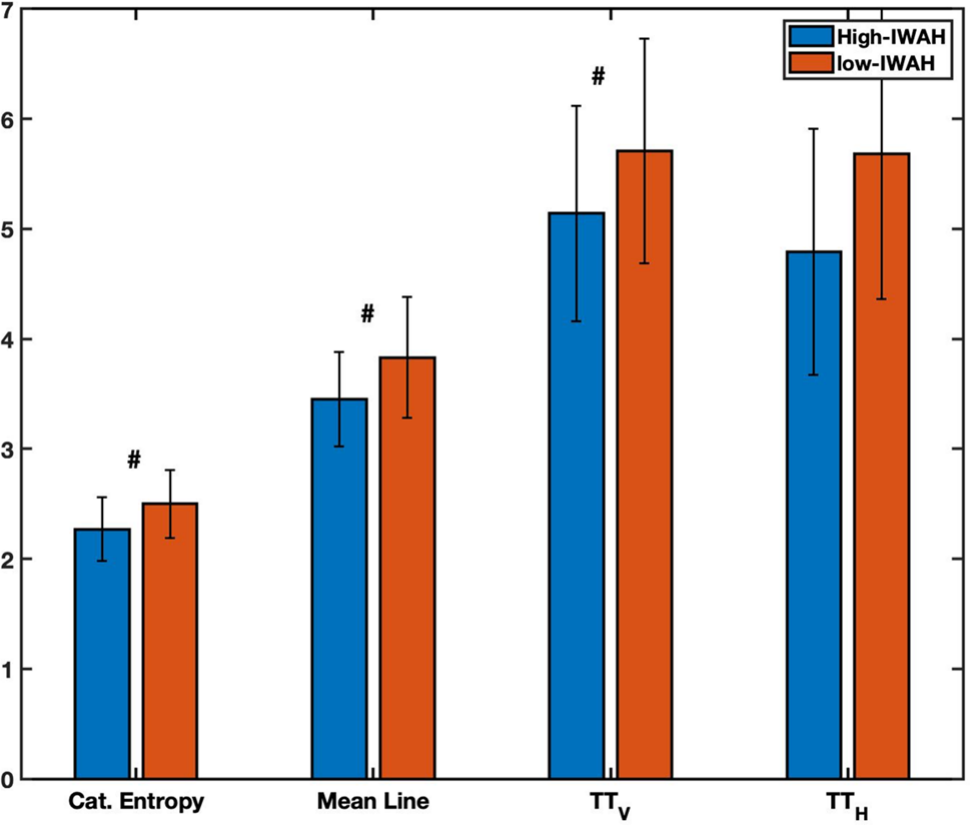
Average CRQA parameters for the high- and low-IWAH groups. Statistical trends (*p* < 0.1) are marked by #.

Finally, we applied Cross-Recurrence Quantification Analysis (CRQA) to investigate the coupling between the movements during the interaction (see the method section for more details). We looked at the total number of recurrences (i.e., RR), the average length of diagonal lines (i.e., MeanLine), the categorical entropy (CatEnt), the trapping time of both vertical (TT_V_), and horizontal lines (TT_H_). Results from the CRQA analysis are presented in Fig. 4. Wilcoxon signed-rank tests showed statistical trends for CRQA parameters (*Z*_MeanLine_ = 1.72, *p* = 0.08; *Z*_TT_V_ = 1.66, *p* = 0.09; *Z*_CatEnt_ = 1.72, *p* = 0.08), except for RR (*Z*_RR_ = 1.12, *p* = 0.26) and TT_H_ (*Z*_TT_H_ = 1.39, *p* = 0.16). The lower CRQA values for the high-IWAH group suggest that the dyadic interaction between them and a lab assistant is more complex and, therefore, more efficient than in the low-IWAH group. These results did not confirm hypothesis 4 but showed statistical trends in the expected direction.

## Discussion

People with higher levels of dispositional identification with all humanity (IWAH) feel a bond with and concern for people all over the world and perceive them as an ingroup^2^. If it is more than just a declaration, we should be able to observe differences in attitudes toward a person of different ethnicity in real interactions, depending on an individual's level of IWAH. The aim of our study was to explore the dynamics of inter-ethnic interactions while performing a collaborative task. We focused on indicators of approach/avoidance tendencies, traditionally treated as manifestations of intergroup attitudes (see, e.g.^6–8^). Instead of using questionnaires to measure intergroup attitudes, for the first time, an approach that combines a state-of-the-art tracking algorithm with a dynamical systems method was applied, using less direct measures, such as spatial distance, coordination, coupling, and leading.

We hypothesized that while performing such a joint task in inter-ethnic dyads, high-IWAH individuals will keep a smaller distance to a partner, take a more leading role, and show better coordination and coupling with a partner than low-IWAH ones. All these hypotheses were confirmed except for coupling, where we still found a statistical trend in the expected direction.

Specifically, our results showed that high-IWAH participants kept closer to a partner of different ethnicity from the start, on average, and during long parts of the 5-min interaction. Thus, spatial distance as a manifestation of intergroup attitude (especially avoidance tendencies) showed that low-IWAH individuals avoided their partners of different ethnicity more than high-IWAH ones. Interestingly, no significant differences were found at the end of the interaction (last 40 s). The analysis of the videos showed that teams were still working on a task, perhaps trying to finish it in time.

Moreover, participants in both groups led the interaction (see Fig. 2), which is not surprising since lab assistants were instructed to be more passive. However, the lag-profile analysis showed qualitatively that during a 5-min joint task, high-IWAH participants were leading longer and in a more stable manner. On one hand, the periods of leading in the high-IWAH group were significantly longer than in the low-IWAH group. On the other hand, the stability is reflected by the presence of a unique peak in the lag profiles of the high-IWAH group, on the part referring to the participant’s leading. However, we observed a drop in participants’ leading in the low-IWAH group, which is indicated by the rise of a peak in the lab assistant leading side of the low-IWAH group. In those situations, an assistant had to take the lead, which might be interpreted as an example of avoidance tendency on the participants’ part. Such a shift in leading was not observed in the high-IWAH group. This suggests that the common goal of the dyad (i.e., building a LEGO construct) was preserved. However, fluctuations of one component (i.e., fluctuations in movements due to the IWAH level of a participant) need to be compensated by fluctuations in the other component (i.e., an assistant taking the lead) to preserve the functional organization of the dyad. This is translated into the qualitative group differences observed in the lag profiles.

Next, we transformed the profiles into density distributions of temporal data (e.g.^21^) and showed quantitatively that there were differences in the leading-following relationships of both groups. Particularly, high-IWAH participants had more effective coordination (ie., higher kurtosis and lower dispersion) and a less symmetric and participant-leading distribution (i.e., right-skewed) in comparison to the low-IWAH group. Altogether, lag profiles proved to be an excellent tool to study the coupling between behaviors and how they influence each other, which here differed due to the identification with all humanity levels.

Also, coordination between dyad partners while performing a task was high in both groups, and both tended to synchronize their body movements at a level significantly greater than chance compared to the random profiles. However, a closer look at the lag profiles shows that there are several differences in the way both groups are coordinated. First, the high-IWAH group displayed a lower recurrence rate (RR) and narrower coordination peaks, which is in line with studies suggesting this as a sign of a more complex and efficient interaction (e.g.^21^). Second, while we did not receive significant differences in coupling between the CRQA parameters, some interesting patterns arose between both groups. Particularly, lower recurrence rate, mean diagonal line, trapping times, and categorical entropy in the high-IWAH group again suggest a more efficient interaction than in the low-IWAH group. This is in line with studies showing that higher complexity is related to lower RQA parameters (e.g.^22,23^). A dyadic interaction lacking structure (i.e., lower complexity) will be less efficient since both interactive partners will not adjust their movements to one another as opposed to a structured and efficient interaction (i.e., high complexity). Additionally, for both groups, the task was new, and participants in both groups needed to equally adapt to the context, which in dynamical systems is often related to higher variability in the complexity of the system (e.g.^17^). The lower CRQA parameters of the high-IWAH group suggest that these participants were able to reorganize into a new stable state faster than low-IWAH, which translates into more efficient coordination with a lab assistant and increased flexibility to reorganize and coordinate. Altogether, we believe that these dynamical measures might shed more light on the unconscious responses displayed during interactions since people could manipulate their answers in surveys by consciously choosing the “proper” ones, but much less—their non-verbal behavior, such as avoidance tendencies or coordination with a partner.

Interestingly, our results were obtained while there were no significant differences between groups in participants' shyness, evaluation of the task, frequency of having friends or acquaintances with different skin colors, and no differences in the strength of such a bond. Therefore, these results suggest that dispositional identification with all humanity affects interactions in inter-ethnic dyads, with people with higher IWAH treating their partners more as ingroup members than ones with lower IWAH do.

Our study has limitations. Given the small sample, however typical for dynamic systems research (e.g.^21,24,25^), future studies should be replicated on bigger, and possibly more diverse populations in different cultural contexts, with a more diverse group of lab assistants, and with control groups. As our group consisted mostly of women, future studies should also aim at more gender-balanced samples of participants. However, the loss of male subjects between stages I and II shows that women were more eager to help and participate in a lab study.

Another limitation is the choice of the radius to categorize the movement. In this paper, we chose a small radius because we sought to study the dynamics of overall body position and wanted to keep as much information as possible. However, this could lead to an over-fitting of the data since a small radius can make time series sensitive to the influence of noise. This is because the camera's sensitivity and DLC test errors are larger than the selected threshold. Additionally, given the area covered by the camera and its resolution, 0.1 pixels could represent a very small movement in the context of human movement. However, DLC did not behave equally in all contexts; sometimes the error was very low, and sometimes it was slightly higher. Second, we reduced the noise in the measurements by smoothing the data which would help decrease those errors. Finally, also in the context of movement, our camera was recording at 30 fps, and it could be that even if the head was moving, the recorded movement was in fact very low. The Euclidean distance of both body parts actually showed that on a frame-by-frame basis the head movements are generally on average below 1 pixel (see Supplementary Section G for examples). Additionally, a larger radius can produce subtle movements to be no longer classified as movements but as the participant being immobile. The size of the video might also influence the choice of a radius, since the same value can provide different results in different camera resolutions. Therefore, future more in-depth analysis is needed to systematically analyze the influence of the radius size in the dynamical measures. Finally, CRQA lag profiles, although informative about who is generally leading the interaction (e.g., a lab assistant, a participant, or both), cannot provide an exact number of how frequently a participant or a lab assistant was leading the interaction.

## Conclusions

As Gipson and colleagues^26^ (p. 194) claim, “coordination with others is fundamental to human activity. Interpersonal coordination is such a natural part of human behavior that it can happen unintentionally … and simply being in the presence of others can cause individuals to act in similar, synchronous ways when they are connected to one another (or “coupled”).” Our study showed that such coordination could be better in inter-ethnic dyads when individuals identify with people all over the world, no matter what the skin color of a partner is and where they come from. People with higher dispositional IWAH engaged in more complex and efficient interaction with persons of a different ethnicity than those lower in IWAH, and they were more active during the interaction, leading longer and in a more stable manner. Our study is the first to demonstrate that individuals who declare that they identify with people all over the world on a higher level indeed show less avoidant tendencies in inter-ethnic dyads and treat their partners more as ingroup members than those with lower levels of this global social identification. Thus, we have demonstrated how such broad identification may change non-verbal behaviors, unconsciously shaping inter-ethnic relationships. However, replication on more diverse and gender-balanced samples is needed.

## Method

The study was approved by The Committee of Ethics in Scientific Research at IPPAS, no 11/VI/2019. All methods were performed in accordance with the relevant guidelines and regulations.

### Participants

227 students from a Polish university took part in a questionnaire stage in exchange for credits for participation: 85.8% of women, aged 18–58 (*M* = 30, *SD* = 9.59). From this group, only 192 students fulfilled the IWAH scale, and from those, we chose 42 participants with the highest (4 and above on a 1–5 scale) and 40 participants with the lowest (2.75 and lower) scores on Identification with All Humanity scale, and after a few months invited them to take part in the laboratory stage of the study. 29 participants accepted the invitation to the lab and were rewarded with additional credits. One person's data was removed from the database due to a recording error during the lab task. The final group of 28 participants in a lab part consisted of 82% of women, aged 19–49 (*M* = 34, *SD* = 9), all “White” Polish nationals (*N* = 16 in the high-IWAH group and *N* = 12 in the low-IWAH group). The student sample was chosen as the most convenient for such time-consuming, two-staged research.

### Procedure

#### The questionnaire part

Participants filled in an online questionnaire, which was a part of a bigger study. Identification with All Humanity scale^1^ was the only one we used for current research to preselect participants for the laboratory stage of the study.

#### The laboratory part

Participants were instructed that the lab study’s topic was memory capacity. Informed consent was obtained from all participants, including an agreement to be recorded while performing a task in a dyad.

To help mask the true purpose of the study, each participant was informed that this part of the study would require them to work on a task with another participant in an adjacent room. It was a necessary masking instruction (a procedure common in this kind of studies), which was debriefed later. It was crucial for our study to keep participants convinced that their interaction partner has exactly the same role; otherwise, we would introduce unwanted additional ingroup-outgroup division. Individuals participated in a study separately, each time paired with a lab assistant (one of two “Black” female lab assistants from Nigeria, students of the same university, playing their roles alternately).

The experimenter led each participant into a room with a table placed at the wall and two chairs on opposite sides. One chair was already occupied by a lab assistant. The experimenter asked a participant to sit at the table (see Drawing 1 in Supplementary Material A for a setup). It was a modified task from^15^ used to measure a spatial distance between interaction partners.

After a participant sat at the table opposite a lab assistant, they had 5 min to build together a LEGO construction to express their understanding of an abstract concept. Each participant drew a concept to work on by choosing one out of three cards lying on the table (blank side up) with the concepts: *knowledge*, *safety*, or *tolerance*. This task was adapted from a study by Fusaroli and colleagues^24^. Then the experimenter left the room, leaving a participant and a lab assistant alone while performing their task. Each lab assistant was instructed to not move the chair during the interaction, to be nice, and let a participant lead the task and step in only if a participant would be passive.

We used a side camera to record each participant entering the room, taking place at the table, and interacting while performing a task. The video was acquired with a resolution of 1280 × 720 pixels at 30 frames per second. The recording was started before a participant entered the room and stopped after the task. After 5 min spent on performing the task, the experimenter entered the room to stop the task and ask a participant to go to another room, where a short online survey was administered. After the study, all participants were thanked and debriefed.

### Measures

#### Identification with all humanity

Identification with all humanity was measured using McFarland et al.’s^1^ nine-item IWAH scale in Polish adaptation (^27^, e.g., "How close do you feel to each of the following groups…" "people all over the world," "How much would you say you care (feel upset, want to help) when bad things happen to…?" "all humans everywhere"). All items used five-point scales, but the anchors differed based on question-wording (e.g., 1—*not at all* to 5—*very close; 1—almost never to 5—very often,* Cronbach’s *α* = 0.89).

#### Shyness

Shyness was measured by the 12-item Revised Cheek and Buss Shyness Scale (RCBS^28^) in a Polish adaptation (^29^; e.g., "I feel tense when I'm with people I don't know well," "It is hard for me to act natural when I am meeting new people," "I do not find it difficult to ask other people for information" (reverse scored) with a 5-point scale from 1—*I do not agree at all* to 5—*I completely agree*. Cronbach’s *α* = 0.88).

#### Dynamics of the interaction

The dynamics of the interaction (spatial distance, leading, coordination, and coupling between participants and a lab assistant) were measured by body movements. We used two tools for these analyses:

##### DeepLabCut

To extract movement from the videos, we followed the approach highlighted in^30^. First, we used an open-source toolbox called DeepLabCut (DLC) that builds on a state-of-the-art pose estimation algorithm (i.e., a computer vision technique that predicts and tracks the location of a person, animal, or object) to precisely track user-defined body parts^31^. DLC allows the user to train an extremely deep neural network using limited training data and estimates the positions of the tracked anatomical landmarks in each video frame independently. Some of the major advantages are that DLC does not need additional technology (e.g., markers in the body of the participant) or any knowledge of the movement being performed, it provides a frame-by-frame description of movement, and is able to make pose estimates despite occlusion of the subject (for further information, see Supplementary Material B and C). Here, we defined six features to be tracked for each participant (i.e., both hands, a nose, an ear facing the camera, one point in the neck, and one point in the trunk; see Supplementary Video. The participant and the lab assistant granted their informed consent to publish their images in this video). One hundred fifty frames were used to train the algorithm (~ 30 min of manual work), which was trained for 1,000,000 iterations. After the training was finished, the algorithm was used to estimate the coordinates for the remaining frames (for an example of how the algorithm tracks the defined body parts, see Supplementary Video). Before the analysis, the data were filtered using a median filter with a window size of 7 to avoid 1-time-point-outliers.

DLC returned a set of coordinates for each tracked body part. In this paper, we focused uniquely on gross body dynamics (e.g., leaning forward or backward), and therefore, we analyzed head movements (i.e., nose). First, to quantify movements of approach or avoidance, we calculated the spatial distance in pixels as the euclidean distance between the x and y coordinates of the head of the participant and the head of the lab assistant. Next, to analyze this data further, each set of coordinates was subsequently translated into a categorical time series indicating the direction of movement using a categorization scheme where we considered three categories of movement (i.e., left, right, and no movement^25^). No movement was considered if the euclidean distance between the one-time point and the next one was below 0.1 pixels. Moreover, given the positioning of the camera, the movement of a participant was mirrored by the movement of a lab assistant (i.e., while approaching the LEGO pieces on the table, a participant was moving to the right, but a lab assistant had to move left). To make the interpretation of the dynamical results easier (see the section below), the time series of a lab assistant was shifted (i.e., left-shifted to right and vice versa). In this way, if a participant and an assistant lean toward LEGO, this would be considered a ‘match.’ The same would happen when both would lean back. All other combinations (e.g., the lab assistant moving forward and the participant leaning back) were considered ‘mismatches.’ This is how we measured coupling.

##### Cross-recurrence quantification analysis

Next, we used Cross-Recurrence Quantification Analysis (CRQA^32^) to quantify the collective dynamics between participants during the LEGO task. Thus, first, we aligned the categorized movement time series extracted from DLC (see the previous section) and applied diagonal-wise CRQA to measure their temporal coupling. Positive matches between time series are represented with a point (i.e., recurrence) in the recurrence plot, which represents the global structure of recurrence^33^. The analysis of recurrences near the main diagonal allows for reconstructing a lag profile, which contains information about the coordination of those time series (e.g.^25^). For each interaction, a diagonal lag profile between − 3 and + 3 s was calculated in Matlab (MATLAB 2019b, The MathWorks, Inc., Natick, Massachusetts, United States) using a translated version of the *drpdfromts* function from the CRQA R-package^34^. The distribution profile was compared to a baseline condition to validate that the patterns within the diagonal profiles are real and do not arise by chance^35^. This baseline was computed as a cross-recurrence of the participant categorized time series and a time series resulting from a random shuffling of a lab assistant’s time series. In our lag profiles, negative values of the profile indicate a participant-leading role while positive values—a lab assistant-leading one. Finally, after computing the lag profiles, we performed a distribution analysis, treating the profiles as density distributions of temporal data (e.g.^21,36^). From those density distribution profiles we extracted the following measures:

- Kurtosis: a measure of how the density distribution peaks. Higher values would indicate a higher concentration of recurrence in a smaller interval of lags (i.e., a more punctual or effective coordination).
- Dispersion: an index of how punctual/effective coordination is. Narrower distributions would be indicated by lower values of standard deviation and would indicate more effective coordination.
- Skewness: a measure of the asymmetry of the distribution. A right-skewed distribution will suggest an interaction led more by the participant, while a left-skewed one would be related to the lab assistant leading more.

In addition to the profile for the entire interaction, we calculated additional CRQA parameters using the *crqa* function:

- Recurrence Rate (RR): it represents the percentage of recurrent states (e.g., the number of times the participant moves to the left after having already moved to the left) and provides a general measure of how well coordinated both participants were.
- Mean Line (MeanLine): it is the average time participants coordinate their movements, and it can be interpreted as the mean prediction time.
- Trapping Time (TT): is measured in units of time and estimates how long subsystems are, on average, *trapped* in a specific state. If TT_V_ (vertical) is high, the participant will be trapped in relatively long periods of the same behaviors that are matched by the lab assistant, and for high TT_H_ (horizontal), a lab assistant will be trapped in relatively long periods of the same behaviors that are also expressed by the participant at some point^37^.

Finally, we also estimated the categorical entropy (CatEnt) from the recurrence plot. CatEnt estimates the frequency distribution of the areas of rectangular recurrence blocks emerging as the prevalent pattern in categorical time series^38^. Higher values correspond to increased variability in the periods in which both participants were coordinated, while lower values are related to more repetitive deterministic patterns and lower variability in the duration of the periods of coordination. It was estimated using an in-house Matlab version of the R function following Leonardi^38^.

#### Evaluation of LEGO task

We used three items to ask for participants' evaluation of LEGO task: how much they liked the task if they were satisfied with the final result, and how easy or difficult it was to reach an agreement with a partner about LEGO project. All items used five-point scales, but the anchors differed based on question-wording (1—*not at all*; 5—*very much* or 1—*very difficult*; 5—*very easy*).

#### Intergroup friendships

Additionally, for control reasons, we asked participants if they have persons with skin color among their friends or acquaintances (1 item) and how close this relationship is (1 item). Both items used five-point scales. This item was important, as Poland is one of the most homogenous countries in the world, predominantly "White" (see more, e.g.^20^), so not all participants would know such a person.

## Supplementary Information

Supplementary Information.

## Data Availability

The data and the scales used are openly available upon request at the first author and at https://iwahlab.com/resources. The video showing an example of how the algorithm tracks the defined body parts can be found here: https://mega.nz/file/Q6QhyQBL#sUQELZdqI6Jq-VrEIjVCguxhR3a2L7UvSp9lgv7ISrg. The Matlab scripts and DLC files used in this manuscript are openly available upon request from the second author.
