# Supplementary material for: The dynamics of dyadic interactions between people of different ethnicities depend on their identification with all humanity
Source: Sci Rep. 2022 Dec 15;12:21671. doi: 10.1038/s41598-022-25905-9 (PMC9755471; doi:10.1038/s41598-022-25905-9)
Supplement: Supplementary file 1 — Supplementary Information. [file 41598_2022_25905_MOESM1_ESM.docx]

**Online Supplementary materials**

**- Supplementary material A**

Drawing 1. An example of a setup during performing the LEGO task in the lab.

**
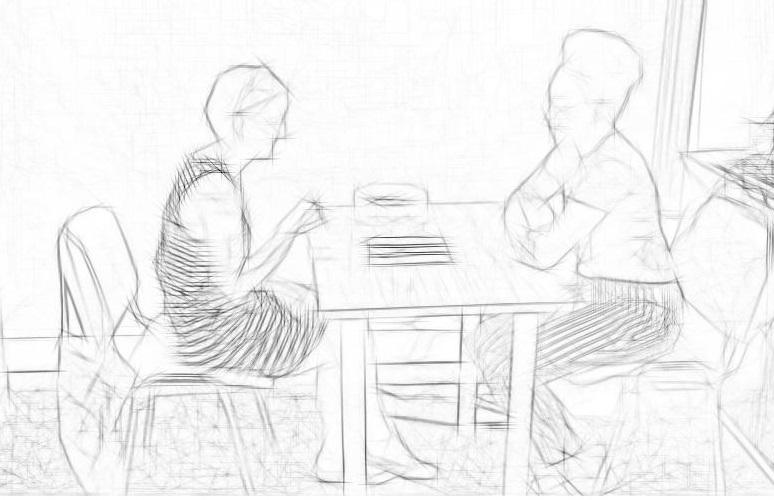
**

- **Supplementary material B**

**Introduction to the tools we used for tracking adult human poses/movements**

Advances in computer vision methods lead to the development of many excellent tools for tracking adult human poses or movements. Computer vision methods rely on deep neural networks to track and predict the location of a person, body part, or object in images and videos. In the current research, we employed a recently developed tool called **DeepLabCut** (DLC; **[1]**) that builds on a state-of-the-art pose estimation algorithm. DLC allows the user to train an extremely deep neural network using limited training data to precisely track user-defined features that match human labeling accuracy **[1]**, which allows estimating the positions of the tracked anatomical landmarks in each video frame independently. Some of the major advantages are that DLC does not need additional technology (e.g., markers in the body of the participant), provides a frame-by-frame description of movement, does not require any knowledge of the movement being performed or information from previously tracked video frames allowing it to potentially track any movement with similar accuracy and it is able to make pose estimates despite occlusion of the subject. To date, only a handful of studies have used DLC to analyze human data (e.g., **[2, 3]**), and to our knowledge, there are no studies using DLC in the context of social psychology (especially of intergroup behaviors).

We combined DLC with the use of dynamical system methods; an approach recently developed to study the online dynamics of interactions **[4]**. Particularly, Cross-Recurrence Quantification Analysis (CRQA) is a method that has gained popularity in psychology to measure coordination between participants in different contexts (e.g., **[5, 6, 7, 8, 9, 10]**). CRQA enables us to capture in precise detail the dynamical aspects of the contributions of the individuals during the interaction and infer the relative role of both partners (e.g., whether leader-follower relationships are in place). Here, we applied CRQA on time series extracted with DLC to study the complex behavior of low- and high-IWAH participants interacting with a lab assistant of different ethnicity to (1) test whether individual movements of both partners are coupled in time and (2) study differences in the quality of this coordination.

- **Supplementary material C**

**The effectiveness of DeepLabCut (DLC) in studying intergroup relations**

It is worth emphasizing that this is, to the best of our knowledge, the first use of DeepLabCut (DLC) in the context of intergroup relations study. DLC proved to be an efficient method to track the movement of participants, showing remarkable accuracy (test error = 4.12 pixels) given the low number of labeled frames (~1.67% of a total number of frames). DLC is yet another computer vision method, among other excellent tools for tracking adult human poses, such as OpenPose **[11]** or DeepPose **[12]**. On the one hand, in comparison to manual coding, DLC is easy to set up, use and less likely to be subjective (see Manual in **[13]**). In contrast, manual coding requires extensive training in the coding scheme, multiple coders, as well as high inter-rater reliability and is very time-consuming **[4]**. On the other hand, compared to other computer vision methods, DLC provides flexibility and allows to label the specific body parts we are interested in rather than fitting a skeleton. It works efficiently when body parts are occluded. Altogether, methods like DLC provide a powerful tool for researchers that wish to robustly track specific limbs **[3]** or body parts of participants during interactions.

**References:**

[1] Mathis, A. at al. DeepLabCut: markerless pose estimation of user-defined body parts with deep learning. *Nature Neuroscience*, **21(9),** 1281–1289. [ttps://doi.org/10.1038/s41593-018-0209-y](https://doi.org/10.1038/s41593-018-0209-y) (2018).

[2] Wei, K., & Kording, K. P. Behavioral tracking gets real. *Nature neuroscience*, **21(9),** 1146-1147. <https://doi.org/10.1038/s41593-018-0215-0> (2018).

[3] Pouw, W., Trujillo, J. P., & Dixon, J. A. The quantification of gesture–speech synchrony: A tutorial and validation of multimodal data acquisition using device-based and video-based motion tracking. *Behavior research methods*, ***52*(2),** 723-740. (2020).

[4] López Pérez, D., Stryjek, R., & Rączaszek-Leonardi, J. Recurrence quantification analysis in the study of online coordination in Norway rats (Rattus norvegicus). *Journal of Comparative Psychology*, **135(1),** 142–149. [https://doi.org/10.1037/com0000253](https://doi.apa.org/doi/10.1037/com0000253). (2021).

[5] Shockley, K., Santana, M., & Fowler, C. A. Mutual interpersonal postural constraints are involved in cooperative conversation. *J. Exp. Psychol. Hum. Percept. Perform.* **29,** 326–332. doi: 10.1037/0096-1523.29.2.326. (2003).

[6] Richardson, D.C., & Dale, R. Looking to understand: the coupling between speakers' and listeners' eye movements and its relationship to discourse comprehension. *Cogn. Sci.* **29,** 1045–1060. DOI: 10.1207/s15516709cog0000_29 (2005).

[7] Shockley, K., & Turvey, M. T. Encoding and retrieval during bimanual rhythmic coordination. *J. Exp. Psychol. Learn. Mem. Cogn.* **31,** 980–990. doi: 10.1037/0278-7393.31.5.980. (2005).

[8] Stephen, D. G., Dixon, J. A., & Isenhower, R. W. Dynamics of representational change: entropy, action, and cognition. *J. Exp. Psychol. Hum. Percept. Perform.* 35, 1811–1832. doi: 10.1037/a0014510. (2009).

[9] López Pérez, D., Stryjek, R., & Rączaszek-Leonardi, J. Recurrence quantification analysis in the study of online coordination in Norway rats (Rattus norvegicus). *Journal of Comparative Psychology*, **135(1),** 142–149. [https://doi.org/10.1037/com0000253](https://doi.apa.org/doi/10.1037/com0000253) (2021).

[10] Ardizzi, M. et al. Audience spontaneous entrainment during the collective enjoyment of live performances: physiological and behavioral measurements. *Sci Rep,* **10,** 3813. <https://doi.org/10.1038/s41598-020-60832-7> (2020).

[11] Cao, Z., Hidalgo Martinez, G., Simon, T., Wei, S.-E., & Sheikh, Y. A. OpenPose: Realtime Multi-Person 2D Pose Estimation using Part Affinity Fields. IEEE Transactions on Pattern Analysis and Machine Intelligence, **1–1**. <https://doi.org/10.1109/tpami.2019.2929257> (2019).

[12] Toshev, A., & Szegedy, C. DeepPose: Human Pose Estimation via Deep Neural Networks. <https://doi.org/10.1109/CVPR.2014.214> (2013).

[13] Nath, T. et al. Using DeepLabCut for 3D markerless pose estimation across species and behaviors. *Nat Protoc* **14,** 2152–2176 <https://doi.org/10.1038/s41596-019-0176-0> (2019).

- **Supplementary material D**

The supplementary video shows an example of how the algorithm tracks the defined body parts. It can be found here: <https://mega.nz/file/Q6QhyQBL#sUQELZdqI6Jq-VrEIjVCguxhR3a2L7UvSp9lgv7ISrg>

Informed consent to publish was obtained from the participants in the Supplementary video, for publishing their images in an open-access online journal.

- **Supplementary material E**

To analyze the distance during an interaction in more detail, we studied the combined positions of a participant and a lab assistant, and the distance between them. Figures E.1 and E.2 show the horizontal position of the participants, lab assistants, and the distance between them for the low- and high-IWAH groups. Figures E.3 and E.4 show separately the horizontal position of the participants and lab assistants respectively. E1 and E2 plots show that the x coordinates in the High-IWAH group have more ups and downs in the signals, while the low-IWAH group is more stable and passive. Plots E3 and E4 further compare the horizontal position of the lab assistants and participants. They show that a lab assistant position is rather constant in both groups. However, participants are closer (higher values) in the high-IWAH group in comparison to the low-IWAH group. We interpret these results as an indicator that distance-wise the differences are due to the participant sitting closer (in the high-IWAH group) or further (in the low-IWAH group), rather than the behavior of lab assistants.


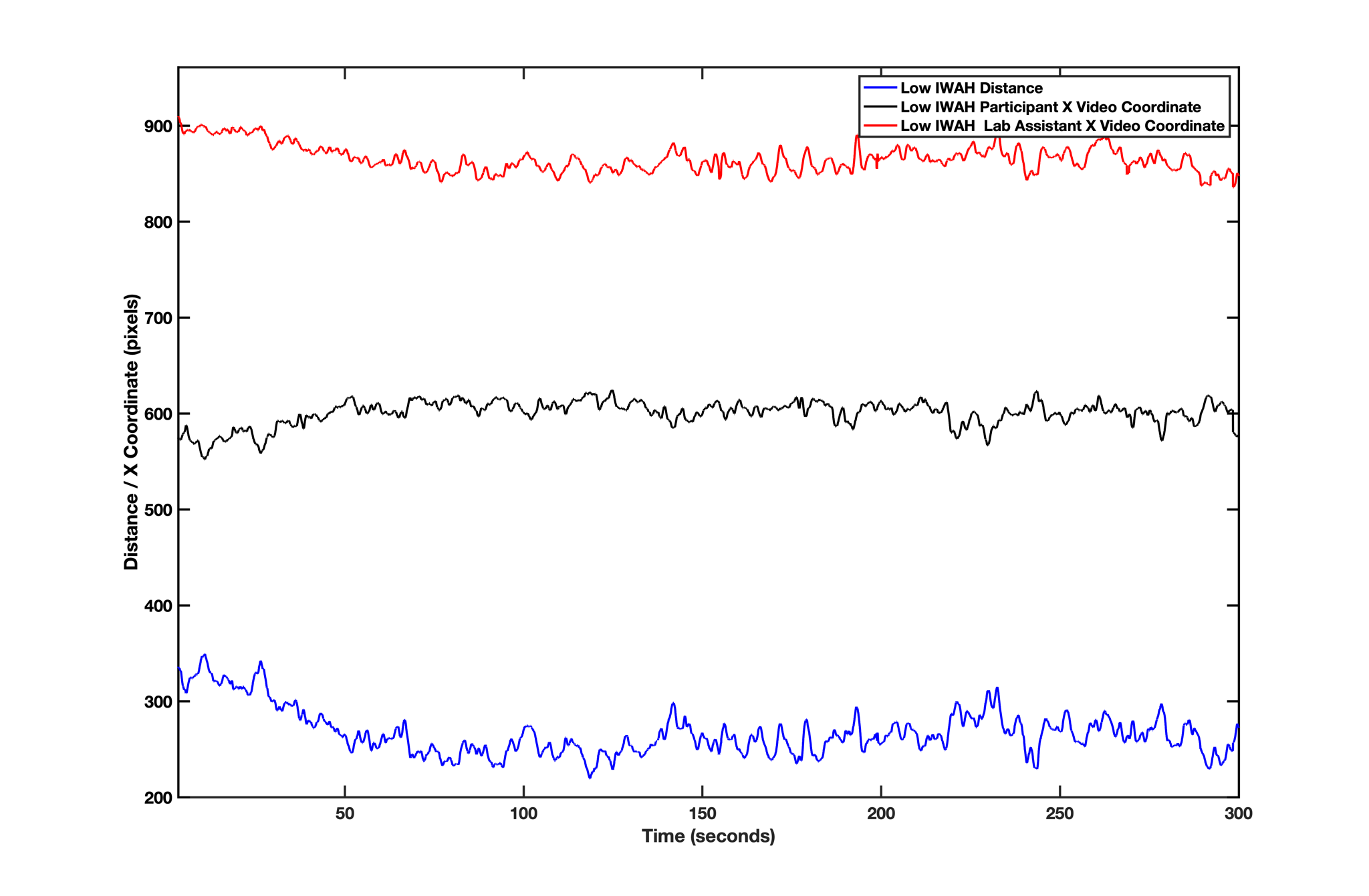


Figure E.1. Average distance between participants and a lab assistant (blue), the horizontal movement of the participant x video coordinate (black), and the horizontal movement of the lab assistant x video coordinate (red) for the low-IWAH group. All measures are in pixels.


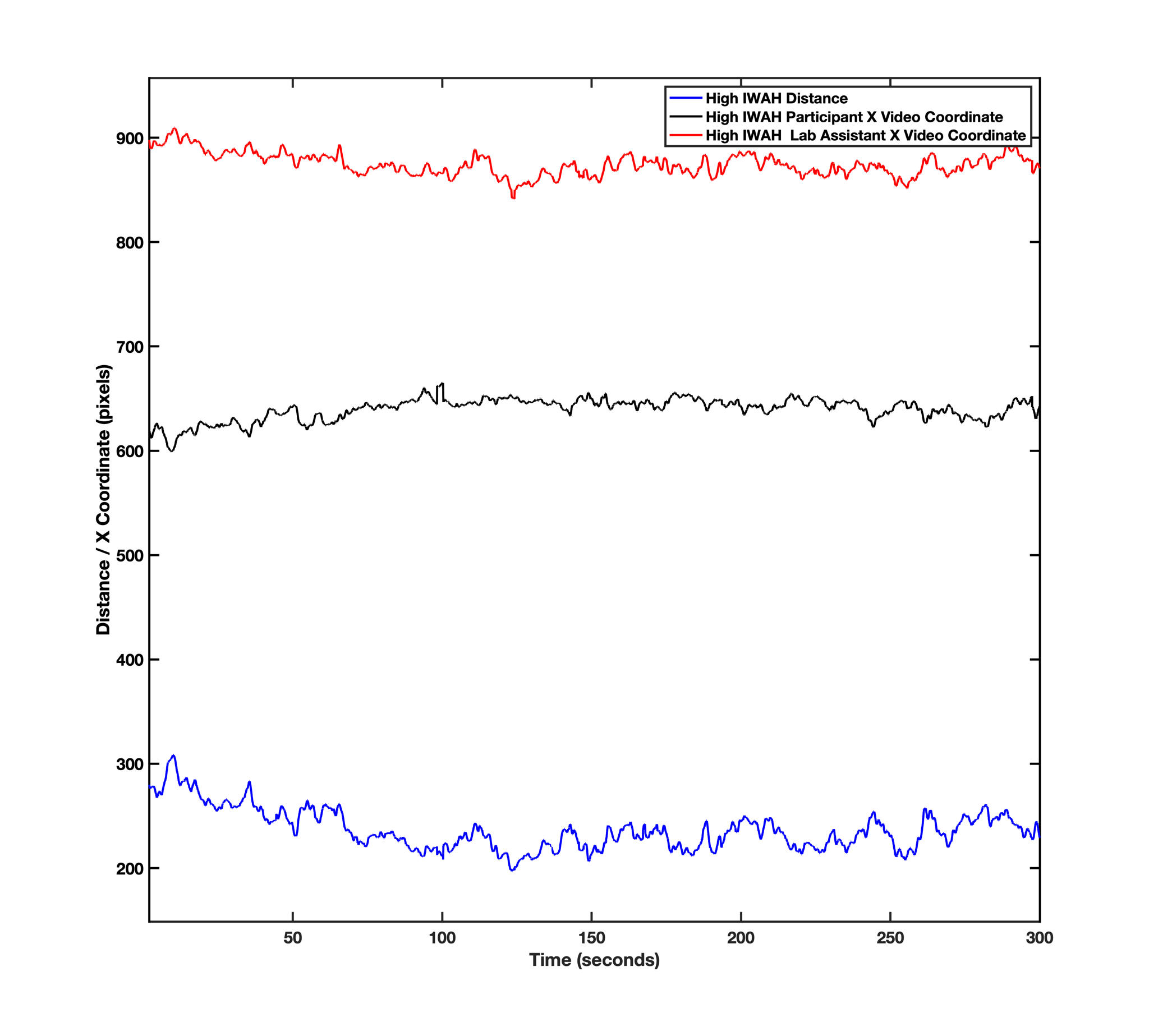


Figure E.2. Average distance between participants and a lab assistant (blue), the horizontal movement of the participant x video coordinate (red), and the horizontal movement of the lab assistant x video coordinate (black) for the high-IWAH group. All measures are in pixels.


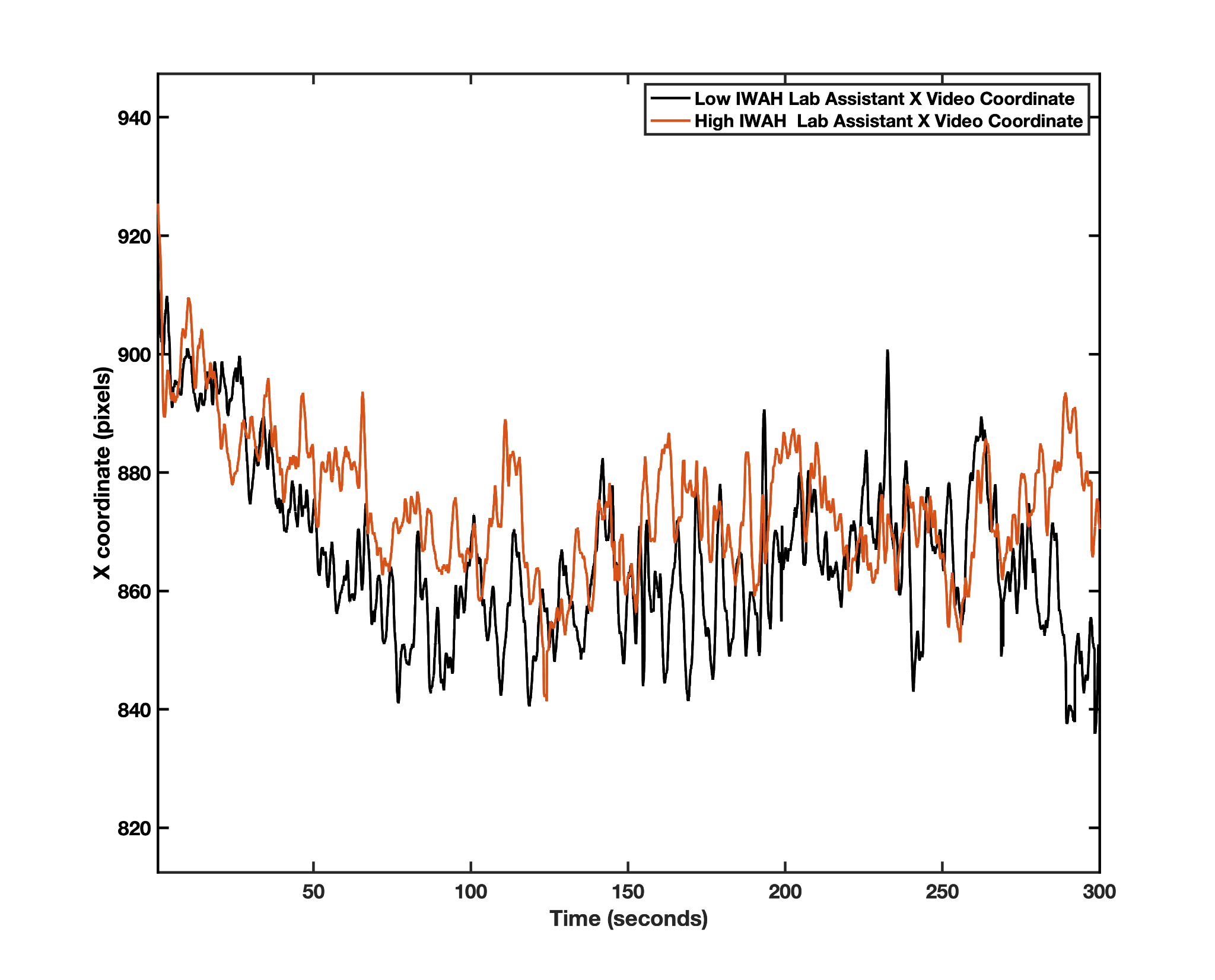


Figure E.3. Horizontal movement of the lab assistants x video coordinate for the low- (black) and the high-IWAH (red) groups.


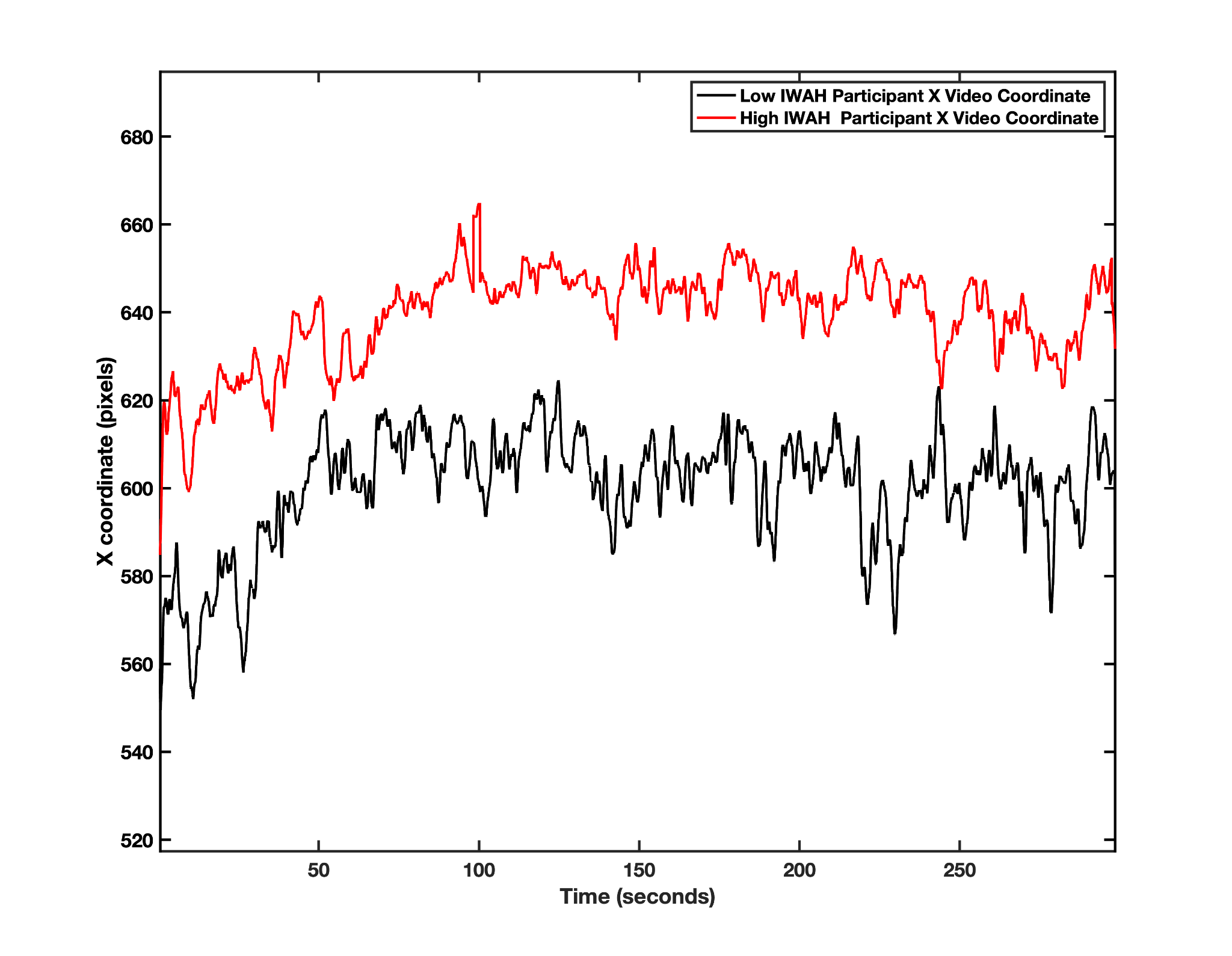


Figure E.4. Horizontal movement of the participant x video coordinate for the low- (black) and the high-IWAH (red) groups.

- **Supplementary material F**

To explore further measures that could help differentiate between “leaders” and “followers” in the interaction, we introduced a measure called lagDiff which is given as the total recurrence in the left  side of the profile minus the total recurrence in the right side of the profile.

lagDiff = totalRRLeft - totalRRRight

The results of such analysis showed no significant differences were present between groups (*ps* > .05) or within groups comparing totalRRLeft vs. totalRRRight (*ps* > .05). This is probably because it is not about the total amount of recurrence but about the shape the profile has around certain lags where coordination arises.

**- Supplementary material G**

Euclidean distance of the head movements of one of the dyads was very low. On average the frame-to-frame Euclidean movement was .92 ± .18 pixels (see Figures G1 and G2 for examples for a random dyad).


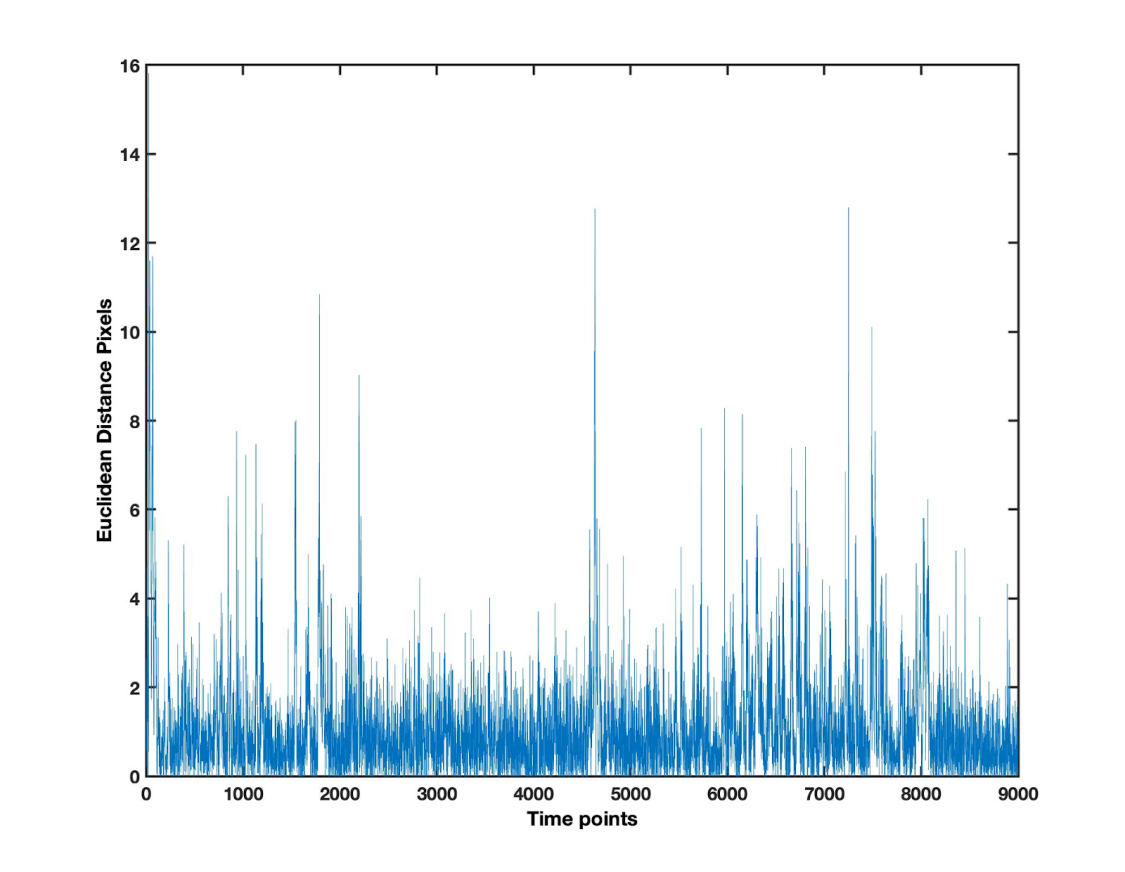


Figure G.1. Frame-to-frame Euclidean distance of the head movements of a participants in a random dyad.


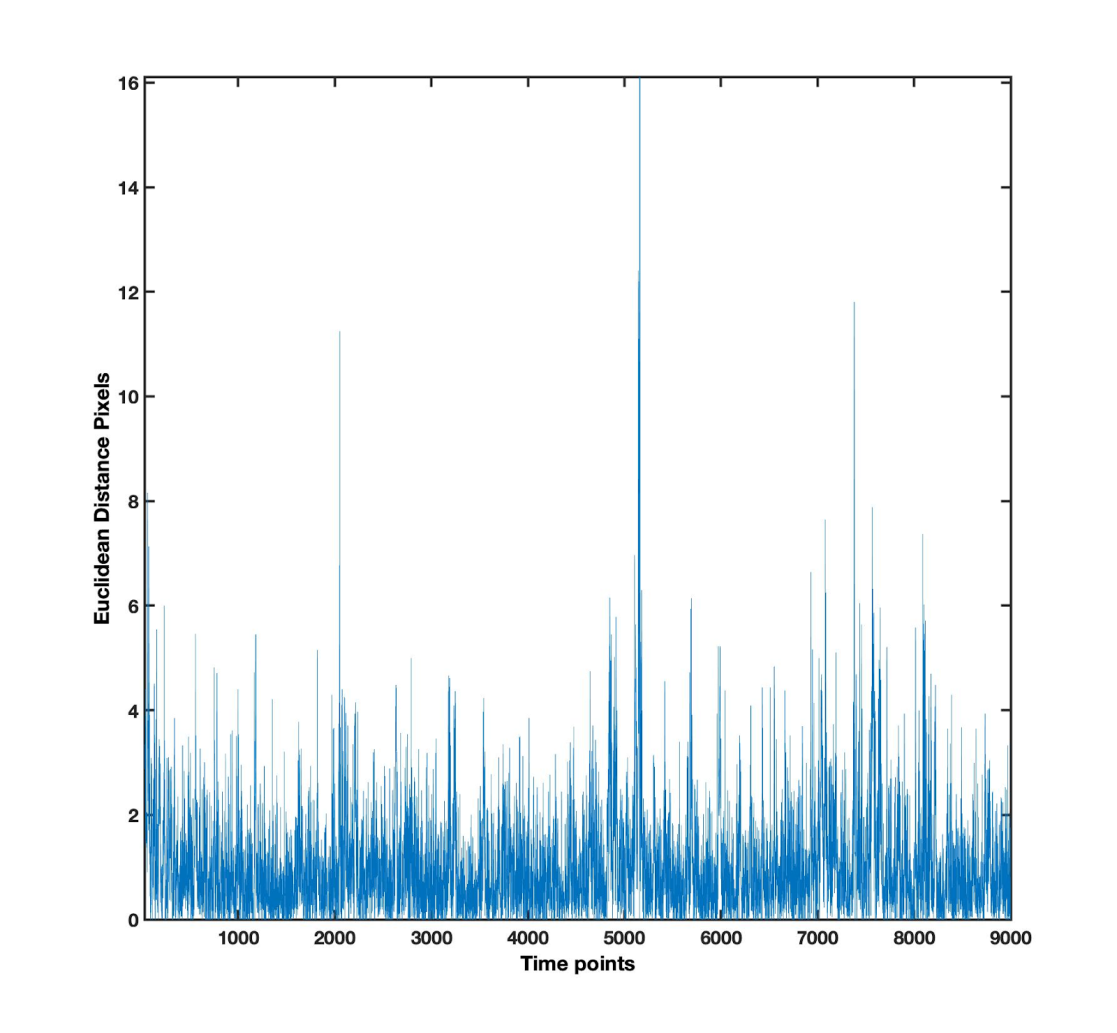


Figure G.2. Frame-to-frame Euclidean distance of the head movements of a lab assistant in the same random dyad presented in G1.

- **Supplementary material H**

Supplementary tables.

| Time Window (seconds) | 0-15 | 15-30 | 30-45 | 45-60 | 60-75 | 75-90 | 90-105 | 105-120 | 120-135 | 135-150 | 150-165 | 165-180 | 180-195 | 195-210 |
| --- | --- | --- | --- | --- | --- | --- | --- | --- | --- | --- | --- | --- | --- | --- |
| Wilcoxon Rank-Test  (Z) | 2.09 | 2.54 | 1.99 | 1.40 | .81 | .71 | 1.94 | 1.35 | 2.14 | 1.85 | 1.60 | 1.35 | 2.34 | 1.80 |
| *p* values | **.04** | **.01** | **.10** | .24 | .42 | .42 | **.03** | .17 | **.03** | **.08** | .15 | .22 | **.04** | .15 |
|  | | | | | | | | | | | | | | |
| Time Window (seconds) | 210-225 | 225-240 | 240-255 | 255-270 | 270-285 | 285-300 |  | | | | | | | |
| Wilcoxon Rank-Test | 1.94 | 2.44 | 1.80 | 1.75 | .22 | -.02 |  |  |  |  |  |  |  |  |
| P values | **.08** | **.02** | **.05** | .15 | 1 | 1 |  |  |  |  |  |  |  |  |

Supplementary Table 1. Wilcoxon Rank-Test comparisons of the distance measures in blocks of 15 seconds. Statistical differences and trends are highlighted in bold.

| Time Window (seconds) | -3 - -2.5 | -2.5 - -2 | -2 - -1.5 | -1.5 - -1 | -1 - -.5 | -0.5 - 0 | 0 - 0.5 | 0.5 - 1 | 1 - 1.5 | 1.5 - 2 | 2 - 2.5 | 2.5 - 3 |
| --- | --- | --- | --- | --- | --- | --- | --- | --- | --- | --- | --- | --- |
| Wilcoxon Rank-Test  (Z) | 2.09 | 2.24 | 2.34 | 1.80 | 1.35 | .86 | 1.75 | 1.85 | 2.88 | 1.94 | 1.60 | 2.04 |
| *p* values | **.03** | **.04** | **.01** | .**07** | .17 | .38 | **.07** | **.06** | **.003** | **.051** | **.10** | **.04** |

Supplementary Table 2. Wilcoxon Rank-Test comparisons of the lab profile in blocks of 0.5 seconds. Statistical differences and trends are highlighted in bold. Negative time window values refer to the left side of the profile, while positive values to the right side.
